# Preventing and countering the interference of tobacco industry: Recommendations from the Joint Action on Tobacco Control 2

**DOI:** 10.18332/tpc/188094

**Published:** 2024-05-16

**Authors:** Renata Solimini, Hanna Ollila, Silvano Gallus, Anne Havermans, Reinskje Talhout, Biljana Kilibarda, Milena Vasic, Esteve Fernández, Dolors Carnicer-Pont, Anna Mar Lopez, Eva M. Pérez-Sacristán, Zsuzsa Cselko, Maurice Mulcahy, Frances O'Donovan-Sadat

**Affiliations:** 1 Istituto Superiore di Sanità, Rome, National Centre on Addiction and Doping, Rome, Italy; 2 Finnish Institute for Health and Welfare, Helsinki, Finland; 3 Department of Medical Epidemiology, Istituto di Ricerche Farmacologiche Mario Negri IRCCS, Milan, Italy; 4 Center for Health Protection, National Institute for Public Health and the Environment, Bilthoven, The Netherlands; 5 Institute of Public Health of Serbia, Dr Milan Jovanovic Batut, Belgrade, Serbia; 6 Tobacco Control Unit, Institut Català d’Oncologia, Barcelona, Spain; 7 Institut d’Investigació Biomèdica de Bellvitge, Barcelona, Spain; 8 Center for Biomedical Research in Respiratory Diseases (CIBERES), Institute of Health Carlos III, Madrid, Spain; 9 School of Medicine and Clinical Sciences, Universitat de Barcelona, Barcelona, Spain; 10 Ministry of Health, Madrid, Spain; 11 Department of Health, Public Health and Food Health TRAGSATEC, Madrid, Spain; 12 National Korányi Institute for Pulmonology, Budapest, Hungary; 13 Health Service Executive, National Environmental Health Service, Galway, Ireland; 14 Head Coordinator, JATC-2, Ministry of Interior and Health, Copenhagen, Denmark

**Keywords:** tobacco industry interference, WHO FCTC Article 5, 3, code of conduct, transparency

The interference of the tobacco industry (TI) in tobacco control policies and activities, is a major reported issue in the European Member States (MS)^1^. The TI continues to oppose effective policies and programs that reduce tobacco smoking and to undermine tobacco control measures, influencing scientific research, politics, law, education, and the media^2^. The Smokefree Partnership recently reported that the TI spent almost €20 million to influence EU policymakers^3^. Moreover, the TI’s cigarette production and marketing directly conflicts with human rights objectives: ‘All people have a fundamental right to breathe clean air and governments are obliged to protect everyone’s health as a fundamental human right’^4^.

The Joint Action on Tobacco Control 2^5^ is an EU funded cooperation project to strengthen policies and actions supporting the implementation of the Tobacco Product Directive (TPD) and the Tobacco Advertising Directive (TAD). Within the activities of Work Package 4 (WP4), ‘Sustainability and cooperation across European MS’, possible measures to counter and possibly eliminate the interference of TI have been explored.

Accordingly, several recommendations have been reported in the ‘Guidance document on how to counteract the interference of TI’^6^, including the signing of a Declaration of Interest (DoI) and the adoption of a Code of Conduct (CoC) specific for government officials and staff involved in tobacco and nicotine control, prescribing the standards with which they should comply in their dealings with the TI.

Related templates are provided in the guidance and the CoC can be personalized by the adopting country that should publish it on the website of the relevant agency/institution/organization, in charge of tobacco and nicotine control policies or activities. Important resources that have been used for the DoI template provided in the guidance are: the World Health Organization (WHO) DoI^7^, and the ‘Toolkit Preventing Tobacco Industry Interference: A Toolkit for Advocates and Policymakers, Based on the Guidelines for the Implementation of Article 5.3 of the WHO Framework Convention on Tobacco Control (FCTC)’, of the Southeast Asia Tobacco Control Alliance (SEATCA), Health Justice^8^. Another important document considered is the ‘Guidance for Public Officials on Interacting with the Tobacco Industry by the Australian Government, Department of Health’^9^. The CoC template was provided by the Global Research and Advocacy at Global Center for Good Governance in Tobacco Control^10,11^ responsible for the Global Tobacco Industry Interference Index^12^.

The recommendations herein provided are a synthesis of the more detailed ones reported in the above-mentioned Toolkit^8^ and in the Australian Guidance for Public Officials^9^, with the exception of the number 11, which is originally created by the authors of the WP4 Guidance^6^. The aim is to support the implementation of Article 5.3 and its guidelines unanimously adopted by the Conference of the Parties of the WHO FCTC in 2008 [decision FCTC/COP3(7)]^13^, which is one of the most important cross-cutting provisions of the Convention, requiring Parties to protect their tobacco/nicotine control and public health policies from commercial and other vested interests of the TI^14,15^.

It is strongly recommended that the governments adopt the following measures:

1. Raise public awareness about tobacco control, TI interference and tactics, and their negative implication for public health.
2. Adopt a CoC based on Article 5.3 FCTC to set common standards for EU officials and public officials/government employees, researchers/scientists of EU countries.
3. With respect to the public officials/government and agencies employees, all interactions with the TI, including front groups that are funded by tobacco and related industries, must be prohibited, unless strictly necessary for regulatory purposes (e.g. the development of law or policy that directly regulates the TI and tobacco/nicotine products). Meeting should be allowed only with stakeholders registered in the EU Transparency Register.
4. Public Agencies and officials must also ensure that staff members are aware of Article 5.3 and monitor any interactions with TI that are out of the ordinary. Transparency of all meetings and interactions with the TI requires that:
  - Detailed information (e.g. the date of the meeting, the organizations represented and a broad description of the issue discussed, related records, minutes, telephone notes, emails, and all communications from tobacco producers and related organizations) must be disclosed on the relevant agency website. For instance:
    - The Netherlands has a protocol of conduct for officials dealing with the TI, a code of integrity that directly references Article 5.3, and a complete disclosure of meetings between officials and the TI. Official communications in the Netherlands regularly reference Art. 5.3^1^;
    - In France TI has to register its lobbying activities in a special registry that is publicly accessible^1^;
    - In Denmark, the Danish Health Agency in order to ensure transparency of the Danish Health Authority’s interaction with the TI, publishes on their website all the communications, inquiries and minutes from tobacco producers and their interest organizations^16^.
  - A minimum of two officials should be present at all times in any meeting or interaction.
  - For email interactions, at least one other official to all communications must be in copy.
  - All meetings or interactions must be recorded and the related information should be comprehensive and detailed.
5. Not allowing any official or employee of the government or of any semi/quasi-governmental body (including local or regional government entities, public, government-owned or publicly supported companies, public universities, etc.) to accept payments, gifts, donations or services, monetary or in kind, from the TI. All the government and semi/quasi-governmental bodies have to be informed by the competent health authorities (national Ministries of Health) about these obligations.
6. Not allowing such official to accept TI contributions on behalf of the government or private entities, and not endorsing, supporting, forming partnerships with, or participating in activities of the TI including activities described as ‘socially responsible’. Indeed, these kinds of activities, including financial contributions to nongovernment organizations, must be denormalized and prohibited.
7. Not allowing TI to work with governments (e.g. to address the illicit trade in tobacco or supporting environmental projects); to promote products purportedly claiming to be less harmful than conventional tobacco products; to provide scholarships or organize or endorse youth or public education initiatives.
8. Preferential tax exemptions, grant incentives, privileges or benefits must not be provided to TI.
9. Declaration of interest (DoI) with respect to the tobacco/nicotine industry to be made before starting a tobacco control relevant project/program or work on tobacco control, and regularly updated in case new interests or changes arise. No organization or individual with a commercial or vested interest in the TI should be involved in developing or implementing public health and related policies/ programs on tobacco control. Any current, previous or proposed connection, involvement or relationship with the TI should be disclosed.
10. Information offered by the TI outside of disclosures required by law, should be carefully scrutinized to minimize opportunities for the TI to manipulate information, cause confusion among the public and government, and undermine public health policies concerning tobacco control. Creating the perception of cooperation between the government and the TI can reinforce the TI’s reputation and generate public acceptance for tobacco companies.
11. Requiring transparency of information from the TI and related organizations (such as the Foundation for Smoke Free World - FSFW), particularly: to provide their annual budget, and to require various organizations receiving money from the FSFW to provide their budget.

Lastly, the WP4 guidance endorses one of the existing recommendations to maximize transparency in connection with the Conference of the Parties (COP) of the WHO FCTC and the Meeting of the Parties (MOP) by summarizing the key decisions FCTC/COP8(12)^17^ and FCTC/MOP1(15)^18^ and guide for participants of COP (FCTC/COP/10/DIV/2/Rev.1) and MOP (FCTC/MOP/3/DIV/2/Rev.1)^19^.

Indeed, this guide for participants nowadays provides clear instructions for the accreditation process for their representatives and ask Parties to indicate that when designating its representatives to the COP/MOP, the Party has observed Article 5.3 of the WHO FCTC and has been mindful of the recommendations 4.9 and 8.3 of the Guidelines for implementation of Article 5.3 of the WHO FCTC^14^.

If all the EU countries implemented the recommendations of this Guidance, signed a DoI, and adopted the CoC, we could avoid waste of financial and human resources to fight against the TI’s aggressive interference at many levels; we could envisage a tobacco and related products-free society with a better optimization of the available resources and improved health conditions of the populations, as well as high economic savings. Furthermore, preventing TI interference on the EU level is pivotal for the timely and forward-looking revision of the directives on tobacco products, taxation and advertising and updated recommendation on smoke-free environments. Recently in its decision from December 2023 (Case OI/6/2021/KR), the EU Ombudsman upheld her finding that the Commission had not been able to ensure a comprehensive approach across all its departments to ensure transparency of meetings with representatives of the tobacco industry^20^. The Ombudsman welcomed the Commission’s commitment to assess the risk of exposure of its departments to tobacco industry lobbying, emphasizing that the European Commission should minimize meetings with the tobacco industry, publicize, and fully minute them^21^. Protection from industry interference on both national and EU level will build capacity and facilitate national actions to progress towards the Tobacco-Free Generation goal of the Europe’s Beating Cancer Plan.

## Data Availability

Data sharing is not applicable to this article as no new data were created.
